# Involvement of the Antioxidant Effect and Anti-inflammatory Response in Butyrate-Inhibited Vascular Smooth Muscle Cell Proliferation

**DOI:** 10.3390/ph7111008

**Published:** 2014-11-10

**Authors:** Omana P. Mathew, Kasturi Ranganna, Shirlette G. Milton

**Affiliations:** Department of Pharmaceutical Sciences, College of Pharmacy and Health Sciences, Texas Southern University, 3100 Cleburne St, Houston 77004, Texas, USA, E-Mails: Mathew_op@tsu.edu (O.P.M.); Milton_SG@tsu.edu (S.G.M.)

**Keywords:** butyrate, vascular smooth muscle cells, glutathione peroxidase, NF-κB, proliferation, inflammation

## Abstract

Epigenetic mechanisms by altering the expression and, in turn, functions of target genes have potential to modify cellular processes that are characteristics of atherosclerosis, including inflammation, proliferation, migration and apoptosis/cell death. Butyrate, a natural epigenetic modifier and a histone deacetylase inhibitor (HDACi), is an inhibitor of vascular smooth muscle cell (VSMC) proliferation, a critical event in atherogenesis. Here, we examined whether glutathione peroxidases (GPxs), a family of antioxidant enzymes, are modulated by butyrate, contributing to its antiproliferation action on VSMC through the regulation of the inflammatory response by using western blotting, immunostaining methods and activity assay. Treatment of VSMC with butyrate not only upregulates glutathione peroxidase (GPx) 3 and GPx4, but also increases the overall catalytic activity of GPx supporting involvement of antioxidant effect in butyrate arrested VSMC proliferation. Moreover, analysis of the redox-sensitive NF-κB transcription factor system, the target of GPx, reveals that butyrate causes downregulation of IKKα, IKKβ, IkBα and NF-κBp65 expression and prevents NF-κBp65 phosphorylation at serine536 causing inhibition of the expression NF-κB target inflammatory genes, including inducible nitric oxide synthase, VCAM-1 and cyclooxygenase-2. Overall, these observations suggest a link between the antioxidant effect and anti-inflammatory response in butyrate-arrested VSMC proliferation, accentuating the atheroprotective and therapeutic potential of natural products, like butyrate, in vascular proliferative diseases.

## 1. Introduction

Vascular smooth muscle cells (VSMC) are highly specialized cells of arterial vessels, and their unintended proliferation in combination with inflammation play a crucial role in the pathogenesis of atherosclerosis [1,2,3,4,5]. Atherosclerosis, a fibroproliferative inflammatory disease of the arterial wall, is responsible for the global burden of morbidity and mortality due to cardiovascular events, such as strokes and heart attacks. Recently, it was acknowledged that many cellular processes that are characteristic of atherosclerosis, such as inflammation, proliferation, migration and apoptosis/cell death, are regulated by epigenetic mechanisms by altering the expression and, in turn, functions of target genes without changing their primary structure. Understanding the epigenetics of atherogenesis and, in particular, its susceptibility to perturbation by epigenetic modifiers, may offer novel insights into the mechanisms of pathogenesis and the assessment of the full potential of epigenetic mechanisms as druggable targets. Although limited epigenetic research has been directed at vascular proliferative diseases [6,7,8,9], our studies and studies by other investigators have shown that epigenetic modifiers, such as histone deacetylase inhibitor (HDACi), including butyrate, arrest VSMC proliferation, an important critical factor in the development of atherosclerosis, restenosis and other vascular proliferative diseases [5,9,10,11]. Butyrate, a natural HDACi and a short-chain fatty acid derived from the colonic microbial fermentation of dietary fiber, displays potential antiatherogenic effect by altering cell cycle regulators and arresting VSMC proliferation [5,9,10].

Besides exhibiting antiproliferation action, butyrate also exhibits an antioxidant effect by augmenting cellular glutathione (GSH), reducing reactive oxygen species (ROS) levels and upregulating several glutathione-s-transferase isoforms (GSTs) in VSMC, implicating the association of a redox component in the antiproliferation effect of butyrate [12]. Moreover, our earlier gene array studies identified the upregulation of several members of the GPx family [13]. GPxs are a family of antioxidant enzymes, which, by scavenging and inactivating hydrogen peroxide and lipid peroxides to water and alcohols, respectively, at the expense of GSH, protect the body from oxidative damage and promote an optimal redox balance in cells [14,15,16,17]. However, increasing evidence indicates that ROS, including hydroperoxides and lipid hydroperoxides, are not only toxic, but also have essential physiological functions [16]. Hydrogen peroxide, which is produced as a byproduct of cellular respiration or intentionally produced by NADPH oxidases in phagocytic leukocytes and in non-phagocytic cells, is utilized for the regulation of detoxification processes and for inflammatory responses [18], cell proliferation [19,20] and signaling [19,20,21]. Studies have shown that, correspondingly, overexpression of GPxs improves protection against oxidative stress and oxidative injury/toxicity in cell culture models and animal models [22,23,24,25,26]. Furthermore, GPxs has been shown to modulate signaling pathways, leading to the activation of a redox-sensitive transcription factor, nuclear factor-kappa B (NF-κB), and, thereby, modulating inflammatory responses [27,28].

NF-κB plays an important role in regulating many cellular processes, including inflammatory and autoimmune responses, cell proliferation and apoptosis, by controlling the expression of genes encoding inflammatory cytokines, cell adhesion molecules and cyclooxygenase-2 (COX-2) [16,29,30]. NF-κB is a key player in inflammation-associated diseases, including cardiovascular disease, cancer and arthritis. A variety of stimuli, including ROS, mitogens and bacteria, are activators of NF-κB [16,31]. Furthermore, NF-κB is the most extensively studied intracellular pathway that is a target of ROS and oxidative stress [16]. Accordingly, neutralization of ROS by the overexpression of GPx causes blocking of the NF-κB cascade, resulting in the attenuation of the inflammatory response by impairing the expression of NF-κB target inflammatory genes [16,27,32,33]. These effects suggest that there is a link between antioxidant and anti-inflammatory response in the regulation of cell proliferation. Even though butyrate has been known to reduce inflammation, as shown in lamina propria macrophages of patients with ulcerative colitis [34] and in colonic biopsy specimens from Crohn’s disease [35], the role of GPxs, particularly the upregulation of GPx4 in the attenuation of inflammatory response by butyrate, is not known, according to our knowledge. Therefore, the present study focuses on confirming the butyrate-induced upregulation of GPxs and their relation to the state of NF-κB in VSMC to determine whether there is an association between antioxidant effect and anti-inflammatory response in establishing the antiproliferation action of butyrate.

## 2. Experimental Section

### 2.1. Materials

The chemicals used were from the following sources: Dulbecco’s modified Eagle’s medium (DMEM), penicillin-streptomycin, trypsin/EDTA and fetal bovine serum from Atlanta Biologicals (Lawrenceville, GA, USA), butyrate from Sigma-Aldrich (St. Louis, MO, USA), antibodies to GPx3 from Novus Biologicals (Littleton, CO, USA) and GPx4 from Rockland Immunochemicals, (Gilbertsville, PA, USA), NF-κB p65, phospho-NF-κB p65 (Ser536), IKKα, IKKβ, IkBα, extracellular signal-regulated kinase 1 and 2 (ERK1/2) and horse radish peroxidase (HRP)-conjugated second antibodies from Cell Signaling Technology (Boston, MA, USA), anti-mouse Alexa Fluor 488, anti-rabbit Alexa Fluor 546 and Hoechst from Molecular Probes (Life Technologies, Grand Island, NY, USA), chemiluminescence luminol reagent from Santa Cruz Biotechnology (Santa Cruz, CA, USA), the micro-BCA protein assay kit from Pierce (Rockford, IL, USA) and glutathione peroxidase assay kit from Cayman Chemicals (Ann Arbor, Michigan, USA).

### 2.2. Cell Culture

Rat VSMC were cultured in DMEM supplemented with 10% fetal bovine serum, 100 units of penicillin and 100 μg/mL streptomycin, which is considered as complete medium. Cells were cultured and maintained in complete medium at 37 ^○^C in a humidified atmosphere with 5% CO_2_, as described previously [12,36]. Confluent cultures were subcultured by splitting the cells at a ratio of 1:6. One day after splitting, proliferating cells were untreated or treated with various concentrations of butyrate for 48 h for a concentration-dependent study. For time-dependent studies, VSMC were exposed to 5 mM butyrate the day after splitting for the required length of time. At this concentration, VSMC proliferation is completely inhibited without any toxicity [12]. At the end of the treatment period, cells were collected for further analysis. All experiments were performed a minimum of three times, unless otherwise mentioned.

### 2.3. GPx Activity Assay

#### 2.3.1. Preparation of Cell Lysate

VSMC cultures were washed with PBS and collected in PBS by harvesting with a rubber policeman. Cell pellets were collected by centrifuging at 2000× *g* for 10 min at 4 °C and homogenized with ice cold buffer containing 50 mM Tris-HCl buffer, pH 7.5, 5 mM EDTA and 1 mM DTT. Cell lysates were centrifuged at 10,000× *g* for 15 min at 4 °C, and supernatants were collected for performing the GPx assay.

#### 2.3.2. Measurement of GPx Activity

The Glutathione Peroxidase Assay Kit from Cayman Chemical (Ann Arbor, Michigan, USA) was used for measuring overall GPx activity in VSMC lysates. The assay kit measures GPx activity indirectly by a coupled reaction with GSH reductase. Overall GPx activity is determined based on the oxidation of GSH to oxidized glutathione disulfide GSSG catalyzed by GPx, which is then coupled to the recycling of GSSG to GSH by GSH reductase and NADPH. The rate of decrease in NADPH absorbance at 340 nm during the oxidation of NADPH to NADP is directly proportional to the GPx activity in the sample. The reaction was carried out in a buffer containing 50 mM Tris-HCl, 5mM EDTA, 1 mM glutathione, 0.4 units/mL of glutathione reductase and 0.2 mM NADPH (pH 7.6) and initiated by the addition of cumene hydroperoxide, which is used as the substrate, as described in the protocol. GPx activities in each sample were measured in triplicate using three different concentrations of cell lysates along with the appropriate no enzyme, no substrate controls and bovine erythrocyte GPx positive control, as recommended by the assay kit. The reaction rate was determined by using the extinction coefficient of NADPH, as described by the manufacturer, and the enzyme activity is expressed as units of activity. One unit is the amount of enzyme that catalyzes the oxidation of 1.0 nmol of NADPH to NADP^+^ per min at 25 °C and normalized to the protein concentration. The data are expressed as specific enzyme activity, units/min/mg protein.

### 2.4. Western Analysis

The whole cell lysates from experimental cultures were prepared in sodium dodecyl sulfate-polyacrylamide gel electrophoresis (SDS-PAGE) sample buffer containing protease and phosphatase inhibitors, as described previously [12,36]. Protein concentrations were determined with the BCA protein assay kit from Pierce Biotechnology (Rockford IL, USA). Samples were denatured by heating in a mixture of 1% β-mercaptoethanol and 0.05% bromophenol blue at 90 °C for 5 min. Equal amounts of denatured proteins were loaded onto polyacrylamide gels for SDS-PAGE. Separated proteins were transferred to a PVDF membrane and probed for immunoblotting with appropriate antibodies [12,36]. ERK1/ERK2 from the same lysate was used as the loading control. Immunodetection was performed with the western blot luminol reagent from Santa Cruz biotechnology (Santa Cruz CA, USA). Band intensities were measured and quantitated by using Molecular Imager FX Pro Plus MultiImager System and Quantity One software (Bio-Rad, Hercules, CA, USA).

### 2.5. Immunofluorescence Staining

Immunofluorescence staining was performed as previously described [12,36]. Cultures were washed three times with PBS and fixed in cold methanol for 5 min. Fixed cells were blocked with 10% heat inactivated horse serum in PBS (HS) for 1 h at room temperature followed by incubation with appropriate primary antibodies in 1.5% HS for 3 h. After incubation, cultures were washed with PBS and incubated with appropriate conjugated second antibody, Alexa Fluor 546 for GPx3 and Alexa Fluor 488 for GPx4 in 1.5% HS for 1 h along with 1 μg/mL Hoechst for nuclear staining. After washing cultures with PBS, images were captured with a Nikon fluorescence inverted microscope with appropriate filters and a CCD digital camera.

### 2.6. Statistical Analysis

Results are expressed as the mean ± SD. Statistical differences between mean values of the groups were determined using one-way analyses of variance (ANOVA) with a Bonferroni multiple comparison test. Statistically significant difference between datasets was determined at *p* < 0.05 to < 0.001. Statistical analysis was performed using Graph Pad Prism version 5 software from Sigma-Aldrich (St. Louis, MO, USA).

## 3. Results and Discussion

### 3.1. Upregulation of GPXs by Butyrate in VSMC

The mammalian GPx family of antioxidant enzymes includes eight different members, GPx1 to GPx8, which exhibit an antioxidant function at different locations and cellular compartments [14,15,16,17,37]. GPx1 in the cytosol and mitochondria, GPx2 in the intestinal epithelium and GPx3 in the plasma function in the aqueous phase; GPx4 present in three different isoforms, cytoplasmic (cGPx4), mitochondrial (mGPx4) and nuclear GPx4 (nGPx4), protects membranes from the oxidative damage of membrane lipids and inhibits the oxidation of lipoproteins. While GPx1 to GPx4 are selenoproteins with selenium at the active center, GPx5 with a cysteine instead of a selenium at the active center is present in epididymis and play a role in male fertility. GPx6 presents as selenoproteins in human olfactory epithelium. GPx7 and GPx8 are also cysteine-containing peroxidases, appearing to play a role in protein folding. Our earlier cDNA array screening studies identified butyrate-induced upregulation of GPx3, GPx4 and GPx5 transcripts [13]. It is surprising that GPx5 transcript is upregulated by butyrate, however, the present study focuses on GPx3 and GPx4, the selenocysteine proteins that have been shown to play protective roles in different inflammatory diseases, including cardiovascular diseases, diabetes and cancer.

#### 3.1.1. Induction of GPx3 Expression by Butyrate

**Figure 1:**
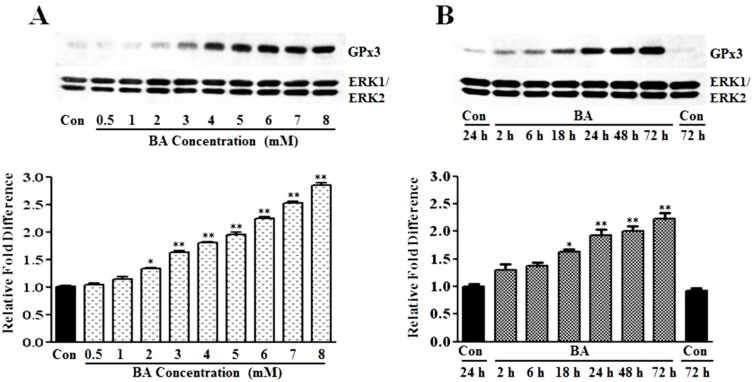
Induction of GPx3 protein expression by butyrate in VSMC. Proliferating VSMC were treated with different concentrations of butyrate for 48 h (**A**) or exposed to 5 mM butyrate for the indicated periods of time (**B**) to determine the concentration- and time-dependent effects of butyrate on GPx3 expression, respectively. At the end of treatment, cell lysates were prepared and processed for western analysis, as described in the Experimental Section. Western blots were probed with anti-GPx3 antibody, and the band intensities of GPx3 were determined and quantitated using an FX Pro Plus MultiImager system and Quantity One Software (Bio-Rad, Hercules, CA). Immunoblotting of ERK1/2 was performed with the same lysate to normalize the protein loading. The results shown are the representative of three independent experiments (**top**). The density of each band is measured and normalized to protein loading. The data are presented as the mean ± SD of three independent experiments and displayed as the relative fold difference compared to the untreated control, (**bottom**). * *p* < 0.05 *versus* untreated VSMC and ** *p* < 0.001 *versus* untreated control VSMC by ANOVA with the Bonferroni test.

**Figure 2:**
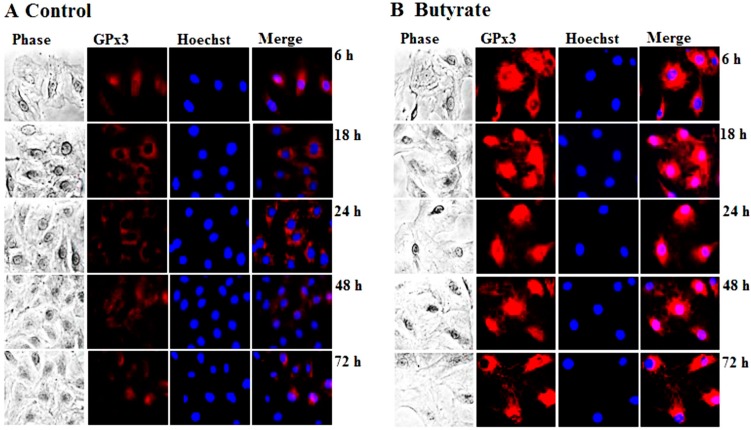
Intracellular localization of butyrate-induced GPx3 expression in VSMC. VSMC untreated (**A**) or treated with 5 mM butyrate (**B**) for different periods of time were fixed and processed for immunostaining of GPx3, as described in the Experimental Section. Images of stained VSMC were captured by a Nikon fluorescence microscope with a CCD camera (400× magnification).

GPx3 is an antioxidant enzyme that plays a role in cellular redox regulation by scavenging and reducing hydrogen peroxide to water at the expense of GSH [14,15,16,17]. Profiling of gene signatures specific to butyrate inhibited VSMC proliferation by cDNA array screening reveals a 3.7-fold increase in the GPx3 transcript compared to untreated VSMC [13]. To confirm whether this increase in transcript level translates to a corresponding increase in GPx3 protein expression, in the present study, the concentration- and time-dependent effects of butyrate on GPx3 protein expression are investigated by western analysis (Figure 1). Although VSMC treated for 48 h with a 0.5 mM to 1 mM butyrate concentration causes no significant effect on GPx3 expression, about a 1.4- to 3-fold increase in GPx3 protein is observed between a 2 mM to 8 mM butyrate concentration in comparison to GPx3 expression in untreated VSMC (Figure 1A). However, no toxicity is observed at any of these concentrations, as reported previously [10,12,36]. Butyrate-induced GPx3 expression is detectable 18 h after 5 mM butyrate treatment, which increases further in a time-dependent manner, reaching about a 2.2-fold increase at the end of 72 h of treatment (Figure 1B). Besides, immunofluorescence staining of VSMC with anti-GPx3 antibody further confirms the upregulation of GPx3 protein by butyrate, not only in cytosol, but also in and around the nucleus in some of the butyrate-treated VSMC (Figure 2).

GPx3 is considered an extracellular enzyme, which is synthesized mainly in the proximal convoluted tubule of kidney and secreted into the plasma [14,15,16,17]. Surprisingly, our present study indicates enhanced cellular expression of GPx3 in VSMC that is localized both in the cytoplasmic and nuclear region in response to butyrate treatment (Figure 2). Besides our study, one other study in bovine mammary epithelial cells indicates cellular localization of GPx3 [38]. Although the significance of the butyrate-induced cellular expression of GPx3 in proliferation-arrested VSMC is not clear, it may contribute to the atheroprotective effect by interfering with VSMC proliferation. Consistent with this, GPx3 has been shown to play a protective role in cardiovascular disease due to atherogenesis [17,39,40]. Furthermore, a relationship between GPx3 levels and the pathogenesis of various other cardiovascular diseases as in arterial thrombosis in ischemic diseases and familial childhood stroke has been reported [41].

#### 3.1.2. Upregulation of GPx4 by Butyrate

**Figure 3:**
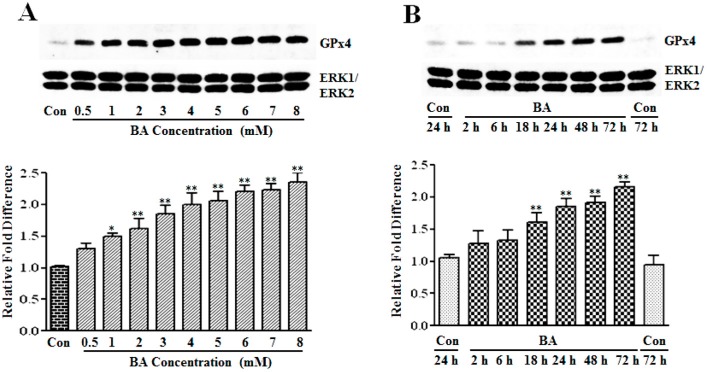
Assessment of the butyrate effect on GPx4 protein expression in VSMC. Proliferating VSMCs were exposed to different concentrations of butyrate for 48 h (**A**) or treated with 5 mM butyrate for the required periods of time (**B**). At the end of the treatment, cell lysates were prepared and processed to determine GPx4 protein expression by western analysis, as described in the Experimental Section. Band intensities were measured, normalized and quantitated, as described in legends to Figure 1. The results shown are representative of three independent experiments (**top**) and the data obtained from three independent experiments are presented as the relative fold difference compared to the untreated control (Con). * *p* < 0.01 *versus* untreated VSMC and ** *p* < 0.001 *versus* untreated VSMC.

**Figure 4:**
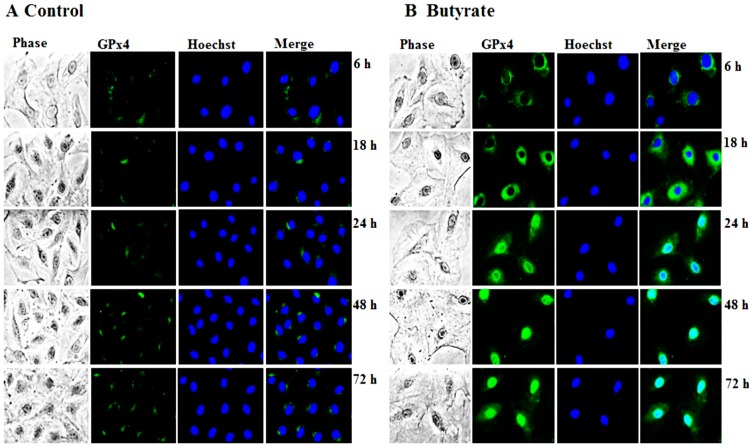
Intracellular localization of butyrate-induced GPx4 expression in VSMC. VSMC untreated (**A**) or treated (**B**) with 5 mM butyrate for different periods of time were processed for immunostaining of GPx4 with an antibody specific to GPx4 followed by Alexa flour 488 conjugated second antibody, as described in the Experimental Section. (400× magnifications).

GPx4, which is also referred to as phospholipid hydroperoxide glutathione peroxidase (PHGPx), is another member of the GPx family that is upregulated in VSMC by about two-fold in response to butyrate treatment in our earlier cDNA array screening studies [13]. To substantiate the induction of GPx4 by butyrate at the protein level, the dose- and time-dependent effects of butyrate on GPx4 protein expression are determined by western analysis. Treatment of proliferating VSMC with butyrate causes an increase in GPx4 protein expression in a concentration (Figure 3A) and time-dependent (Figure 3B) fashion compared to untreated VSMC. Even though the expression of GPx4 is low at 0.5 mM butyrate, about a 2- to 2.5-fold induction of GPx4 expression is observed between a 3 mM to 8 mM concentration range of butyrate at the end of a 48-h treatment (Figure 3A). Significant induction of GPx4 is observed after 18 h of treatment with 5 mM butyrate, which continues to increase all through the experimental period of 72 h. At the end of 72 h treatment, about a two-fold induction of GPx4 expression is stimulated by 5 mM butyrate (Figure 3B). Moreover, intracellular immunostaining of VSMC not only confirms increased expression of GPx4 protein by butyrate, but also reveals localization of GPx4 protein mainly in the cytoplasmic region up until 18 h of treatment. After 18 h of butyrate treatment, a large portion of the butyrate-induced GPx4 is confined to the nuclear region compared to untreated VSMC (Figure 4).

GPx4, a multifunctional 20–22 kDa monomeric selenoprotein, is considered as a key player in important biological contexts, including: function in male fertility, essential for murine embryogenesis, regulation of apoptosis and inhibition of cell proliferation [15,16,17,42]. Importantly, GPx4 is a unique antioxidant enzyme with a distinct ability to reduce lipid hydroperoxides, such as oxidized phospholipids and cholesterol hydroperoxides present in cell membranes and oxidized lipoproteins that are highly atherogenic, besides reducing H_2_O_2_ and small hydroperoxides [16,17,43]. Adding to its unique scavenging activity, GPx4 is also an atypical enzyme with three different isoforms, cGPx4, mGPx4 and nGPx4 forms, but all of them are derived from a single gene organized sequentially [42,44].

The findings of our present study (Figure 3 and Figure 4) clearly indicate the upregulation of GPx4 by butyrate, authenticating our earlier cDNA array analysis [13]. Interestingly, qualitative immunostaining results disclose a time-dependent increase in nuclear localization of GPx4 (Figure 4). Although it is intriguing, at this point, we are not sure whether it is nGPx4 and, if so, what its role is in butyrate-arrested VSMC proliferation. Because, unlike in spermatocytes, where nGPx4 contributes to the condensation of chromatin, in VSMC, butyrate stimulates histone acetylation promoting chromatin decondensation [10,15,17]. Further assessment is needed to confirm the identity of the protein in the nucleus that reacts with the anti-GPx4 antibody to establish its role in butyrate-arrested VSMC proliferation.

Overwhelming evidence indicates that atherogenesis is associated with oxidative stress and mediated by peroxide-induced oxidative modifications of membrane lipids and lipoproteins. Studies indicate that H_2_O_2_ released from the vascular cells oxidize low density lipoprotein (LDL) and its lipid components, which not only induces atherogenic events, such as injury to vascular cells, stimulation of interactions between inflammatory and endothelial cells and induction of VSMC proliferation [45], but also increases the sensitivity of vascular cells to oxidized lipids by triggering oxidative stress-mediated signal transduction pathways, leading to upregulation of a variety of pro-inflammatory cytokines and other proteins. These factors appear to be involved in the recruitment of inflammatory cells to the vessel wall and in the proliferation and death of vascular cells [32,43,45,46,47]. However, overexpression of GPx4 has been shown to protect vascular cells against oxidants and cytokine-mediated inflammatory responses [16,32,47] and against oxidative stress-induced apoptosis [48] and to suppress atherogenesis in apolipoprotein E^−/−^ knockout animals [43]. The results of our present study appear to indicate that upregulation of GPx4 in butyrate-treated VSMC contributes to the arrest of VSMC proliferation by blocking oxidative stress and calming down ROS-mediated signal transduction pathways by scavenging ROS. Supporting this possibility, our earlier study has shown that butyrate treatment of VSMC not only reduces ROS levels, but also increases cellular GSH level and upregulates several isoforms of glutathione-S-transferases (GSTs), which further strengthens the link between the antioxidant effect and the inhibition of VSMC proliferation [12].

#### 3.1.3. Induction of GPx Catalytic Activity by Butyrate

**Figure 5:**
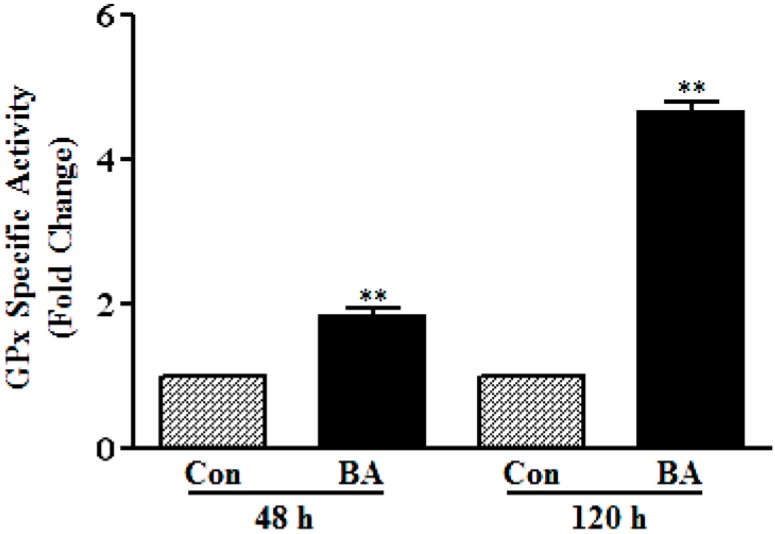
Butyrate induced GPx activity in VSMC. At the end of the indicated treatment period with butyrate (BA), VSMC were washed with PBS and harvested for preparing cell lysates to measure GPx activity, as described in the Experimental section. The data are expressed as the fold difference in the specific activity of glutathione peroxidase. The results are displayed as the mean ± SD. ** *p* < 0.001 *versus* respective untreated VSMC (Con).

To determine whether increase in the protein levels of GPx3 and GPx4 reflects in the corresponding increase in catalytic activity of GPxs, overall glutathione peroxidase catalytic activity is measured (Figure 5). As illustrated in Figure 5, about a 2.0-fold increase in GPx activity is observed in VSMC treated with butyrate for 48 h, which further increased to about five-fold at the end of 120 h of treatment compared to respective untreated controls. Although GPx activity is increased both in 48-h and 120-h butyrate treated VSMC, the increase in GPx activity is not exactly reflecting the increase in expression of GPx3 and GPx4, probably because the assays are performed with crude cell extracts. However, an overall increase in GPx activity distinctly indicates functional upregulation of GPx by butyrate, corroborating the increase in catalytic activity associated with the increase in the expression of GPx isoforms, both at the transcript [12] and protein level.

All in all, the results presented in Figure 1, Figure 2, Figure 3, Figure 4 and Figure 5 collectively appear to indicate that butyrate exhibits antiatherogenic potential by arresting VSMC proliferation by modulating the cellular redox state via upregulation of GPx3 and, particularly, GPx4, the scavengers of proatherogenic ROS along with the upregulation of several isoforms of GSTs and the increase in cellular GSH level [12]. Supporting our data, overexpression of GPx4 has been shown to alter the proliferative response of smooth muscle cells to oxidized LDL, reduce their sensitivity to the hydroperoxide-induced cytotoxicity, apoptosis, block the NF-κB-mediated inflammatory response [16,47], inhibit basal and interleukin-induced VCAM-1 expression [32] and suppress atherogenesis, implicating that the overexpression of GPxs offers protection against the pathogenesis of atherosclerosis [43].

### 3.2. Influence of Butyrate Treatment on NF-κB Pathway and Its Targets

ROS activate the ubiquitous NF-κB transcription factor system, which plays a central role in regulating inflammatory responses, cell proliferation and cell death by modulating the expression of genes [49,50,51,52]. The core components of the NF-κB pathway are the inhibitor IkB kinase complex (IKK complex), the inhibitor IkB proteins and NF-κB dimers. Activation of NF-κB is tightly regulated by its interaction with inhibitory IkB proteins. In most resting cells, homo or hetero dimers of NF-κB are normally sequestered and inactive in the cytoplasm by members of the IkB family of proteins, such as IkBα, IkBβ, IkB€, p105 and p100. Activation of NF-κB by inducing stimuli is achieved through the action of the IKK complex consisting of two catalytic subunits (IKKα and IKKβ) and a regulatory/adaptor protein, IKKγ, also known as NEMO, to form a trimolecular complex [49,50,51,52,53,54]. Activation of IKK complex, a master regulator of NF-κB, by upstream signaling pathways causes immediate site-specific phosphorylation, ubiquitination and degradation of IkB proteins, causing the release of sequestered NF-κB dimers. The released NF-κB dimers translocate to the nucleus, where recruitment of NF-κB to its target genes and regulation of NF-κB-mediated transcriptional activations are facilitated by the phosphorylation of NF-κBp65 at serine536 by IKK [49,50,51,52,53]. Since oxidative stress mediated by ROS activates the NF-κB transcription factor, relevantly, butyrate-induced GPxs, particularly GPx4 overexpression, should block NF-κB activation and NF-κB-mediated proliferative and inflammatory responses through their antioxidant effect. To test whether increased expression of GPx3 and GPx4 in butyrate-treated VSMC alters NF-κB-mediated responses, the effect of butyrate on the status of core components of the NF-κB pathway and the impact on NF-κB gene targets is investigated.

#### 3.2.1. Butyrate Treatment Causes Inhibition of NF-κBp65 Expression and Activation

**Figure 6:**
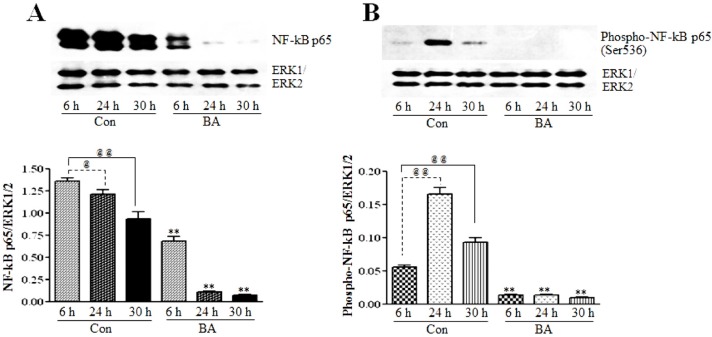
The effect of butyrate on NF-κBp65 protein expression and transcriptional activation. VSMC were treated with 5 mM butyrate for the required periods of time. At the end of the treatment, cell lysates were prepared and processed for assessing total NF-κBp65 and NF-κBp65 phosphorylated at serine536 by western blotting (**top**). Band intensities are normalized to ERK1/ERK2 and presented as a bar graph (**bottom**). Respective data are presented as the mean ± SD of at least three independent experiments. (**A**) Total NF-κB expression is determined by NF-κBp65 antibody (**top**). ^@^ *p <* 0.05 *vs.* 6 h control, ^@@^ *p <* 0.001 *vs.* 6 h control, ** *p* < 0.001 *vs.* respective controls. (**B**) The phospho-NF-κBp65 level is evaluated by the antibody specific to serine536-phosphorylated NF-κBp65. **^@@^** *p <* 0. 001 *vs.* 6 h control, ** *p <* 0.001 *vs.* respective untreated controls.

To determine whether butyrate upregulated GPxs have any association with the position of NF-κB in VSMC, the effect of butyrate on the NF-κBp65 subunit is assessed. NF-κB inhibitors generally inhibit the activation of NF-κB. Surprisingly, even though untreated VSMC exhibit a time-dependent reduction in the NF-κBp65 level, treatment of VSMC with butyrate greatly reduces NF-κBp65 levels compared to their respective untreated controls. This indicates that butyrate treatment inhibits the synthesis of NF-κBp65 in VSMC (Figure 6A).

It is well established that the release of NF-κB dimers from IkB is required for the activation of NF-κB, but it is not sufficient for the full activation of NF-κB-mediated transcriptional activation of target genes. In addition to its release from IkB, several other regulatory steps, including posttranslational modifications, are required for the full activation of NF-κB to stimulate the transcriptional activation of its target genes [49,50,51,52,53]. Phosphorylation of the NF-κBp65 subunit at serine536 by activated IKK in the nucleus is the main posttranslational modification that facilitates the recruitment of the p50/p65NF-κB dimer to the promoter sites of its target genes and regulate their transcriptional activation. To determine whether there is any transcriptionally active NF-κB present in butyrate-treated VSMC, western analysis is performed to detect the phosphorylated NF-κBp65 subunit at serine536. As expected, no visible NF-κBp65 phosphorylated at serine536 is detected in butyrate-treated VSMC, irrespective of the treatment period. This effect is in accordance with the inhibition of NF-κBp65 expression by butyrate (Figure 6A).

#### 3.2.2. Butyrate Treatment Downregulates IKKα and IKKβ and Blocks IkBα Expression in VSMC

**Figure 7:**
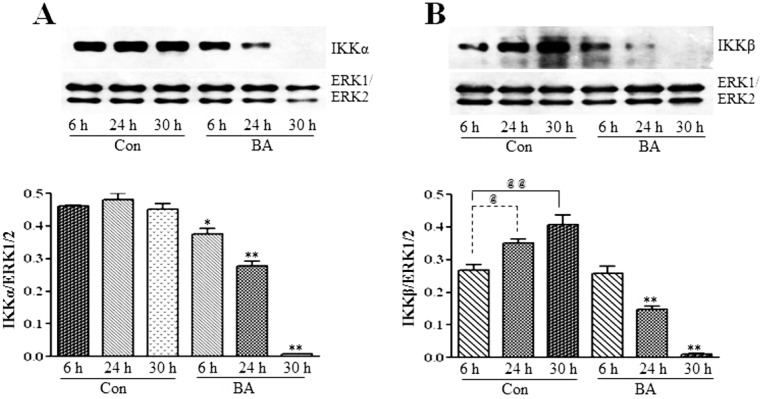
Butyrate downregulates IKKα and IKKβ. VSMC were untreated (Con) or treated with 5 mM butyrate (BA) for the indicated periods of time. At the end of the treatment period, cell lysates were prepared and processed for western analysis. Band intensities are normalized to ERK1/ERK2 and presented as a bar graph in the bottom panels. Values are presented as the mean ± SD of three independent experiments. (**A**) IKKα expression is detected by using anti-IKKα antibody. * *p <* 0.01 and ** *p <* 0.001 against respective controls (Con). (**B**) IKKβ protein expression is evaluated by using anti-IKKβ antibody. **^@^** *p* *<* 0.01 *vs.* 6 h control, **^@@^** *p <* 0.001 *vs.* 6 h control, ** *p <* 0.001 *vs.* respective untreated controls.

**Figure 8:**
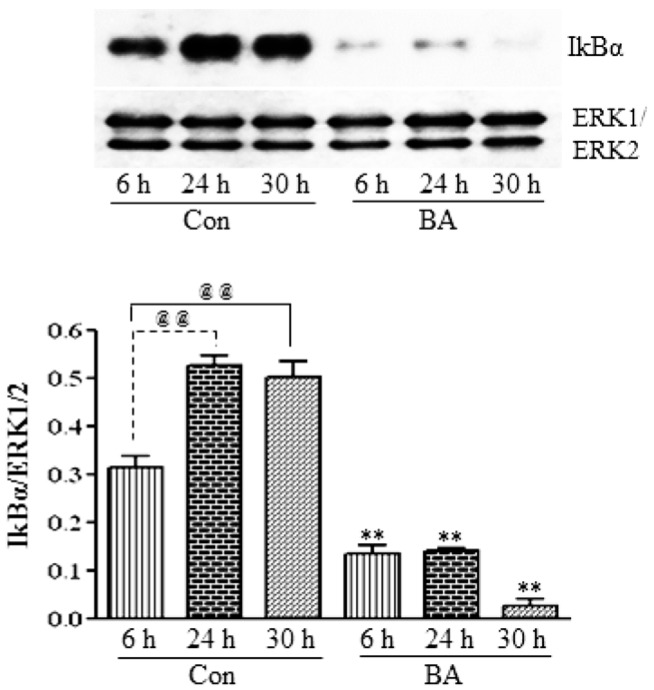
Butyrate inhibits IkBα protein expression in VSMC. Cells were exposed to 5 mM butyrate for the required periods of time and then processed for western analysis, as described in the Experimental Section, to evaluate the effect of butyrate treatment on IkBα protein expression by using anti-IkBα antibody (**top**). Band intensities are normalized to ERK1/ERK2 and displayed as a bar graph (**bottom**). Values shown are the mean ± SD of three independent experiments. **^@@^** *p < 0.001 vs.* 6 h control (Con), ** *p < 0.001 vs.* respective untreated controls.

To investigate whether the inhibition of the expression and activation of NF-κBp65 in butyrate-treated VSMC (Figure 6A,B) is linked to changes in IKKs and IkB, the effect of butyrate on IKKα and IKKβ (Figure 7) and IkBα (Figure 8) is determined. As shown in Figure 7, treatment of VSMC with butyrate causes time-dependent inhibition of the expression of IKKα and IKKβ compared to their respective untreated controls. On the other hand, while IkBα expression in untreated VSMC is increased with the increase in time, its expression in butyrate-treated VSMC is significantly reduced irrespective of the treatment period (Figure 8). These results indicate that butyrate treatment downregulates the expression of IKKα, IKKβ and IkBα, which are required for the activation of the NF-κB dimer-mediated signaling pathway.

It is interesting that the results presented in Figure 6, Figure 7 and Figure 8 collectively indicate that butyrate is inhibiting the NF-κB pathway principally by inhibiting the synthesis of core components of the NF-κB pathway, even though almost all NF-κB inhibitors, including chemopreventive nutraceuticals [54,55,56,57] and butyrate in other studies, inhibit the activation of the NF-κB pathway [34,35,57]. Furthermore, downregulation of components of the NF-κB pathway, particularly in butyrate-treated VSMC that express an increased amount of GPx3 and GPx4, is puzzling, because many studies indicate that overexpression of GPxs inhibits the activation of NF-κB [54,55,56,57]. It is possible that the increase in the GSH level and upregulation of GSTs [12] combined with the upregulation of GPx3 and GPx4 (Figure 1, Figure 2, Figure 3, Figure 4 and Figure 5) in butyrate-treated VSMC collectively reduce the cellular ROS level [12], thus minimizing the need for the NF-κB pathway, resulting in downregulation of components of the NF-κB pathway. Besides our studies, a study that has applied for a patent also indicates the inhibition of the synthesis of NF-κB by natural compounds, such as isoflavones daidzein and daidzin (Patent Application Number EP 2590646 A1).

#### 3.2.3. Attenuation of NF-κB Targets in Butyrate-Treated VSMC 

**Figure 9:**
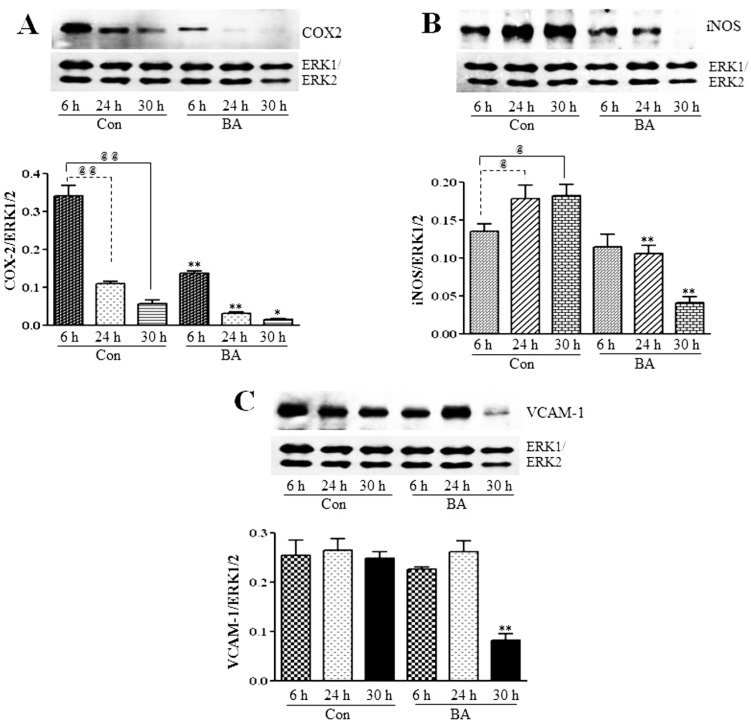
Inhibition of COX2, iNOS and VCAM-1 expression in butyrate-inhibited VSMC proliferation. After the indicated periods of treatment with (BA) or without (Con) butyrate, cell lysates were prepared and analyzed by western analysis to determine the effect of butyrate treatment on the expression of indicated proteins associated with inflammation. Band intensities are normalized to ERK1/ERK2 and displayed as a bar graph, respectively (**bottom**). Values shown are the mean ± SD of three independent experiments. (**A**) COX2 expression. **^@@^** *p* < 0.001 *vs.* 6 h control, * *p* < 0.01 and ** p < 0.001 *vs.* respective controls. (**B**) iNOS expression. **^@^** *p <* 0.01 *vs.* 6 h control, ** *p* < 0.001 *vs.* respective controls. (**C**) VCAM-1 expression. ** *p* < 0.001 *vs.* respective untreated controls.

In many inflammatory diseases, including atherosclerosis, NF-κB is found to be chronically active, playing an important role in the regulation of the expression of a variety of its target genes, including those encoding cytokines, chemokines, adhesion molecules, such as VCAM-1, and inflammatory enzymes, iNOS and COX-2 [16,29,30]. To determine whether the downregulation of components of the NF-κB system by butyrate reflects in the altered expression of NF-κB target genes that are important in the pathogenesis of atherosclerosis, expression of COX-2, iNOS and VCAM-1 is assessed in butyrate-treated VSMC (Figure 9). Even though expression of all three inflammatory proteins is inhibited by butyrate treatment, COX-2 and iNOS expression is attenuated all through the treatment period compared to the respective untreated controls (Figure 9A,B); a significant reduction in VCAM-1 expression is observed only after 30 h of treatment with butyrate (Figure 9C). These results reiterate that butyrate, by inhibiting the synthesis of the components of the redox-sensitive NF-κB pathway displays an anti-inflammatory response by downregulating the expression of COX-2, iNOS and VCAM-1. In contrast, most of the other studies, including studies on VSMC, indicate the inhibition of the expression of NF-κB target genes that contribute to inflammation, such as COX-2, iNOS, VCAM-1 and other inflammatory and proinflammatory proteins by NF-κB inhibitors, resulting principally from the suppression of the transcriptional activation of NF-κB [16,27,32,33,47,55,56]. Furthermore, several reports indicate that even the anti-inflammatory effect exhibited by butyrate by attenuating the expression of COX-2, ICAM, VCAM-1 and the release of proinflammatory cytokines [34,57,58] and reducing inflammation in patients with ulcerative colitis [35] principally involves the suppression of NF-κB activation. Unlike these studies, our present study in VSMC indicates that butyrate exhibits the anti-inflammatory response by downregulating NF-κB target genes COX-2, iNOS and VCAM-1 expression, but their downregulation is mainly linked to the inhibition of the synthesis of core components of the NF-κB pathway. Although we have no explanation for why specifically in VSMC butyrate is causing the downregulation of NF-κB core components, since butyrate is a chromatin modifier and an HDAC inhibitor [5,9,10], it is possible that butyrate may alter the chromatin structure in such a way that promoter sites of the core components of the NF-κB pathway may not be accessible for transcriptional co-activator complexes to turn on their expression, thus inhibiting their synthesis. Further studies are warranted to characterize butyrate’s effect on promoter sites of IKKs, IkB and NF-κBp65 to exploit the utilization of butyrate, in particular, and HDAC inhibitors, in general, to modulate epigenetic mechanisms as an approach to target atherosclerosis.

## 4. Conclusions

**Figure 10:**
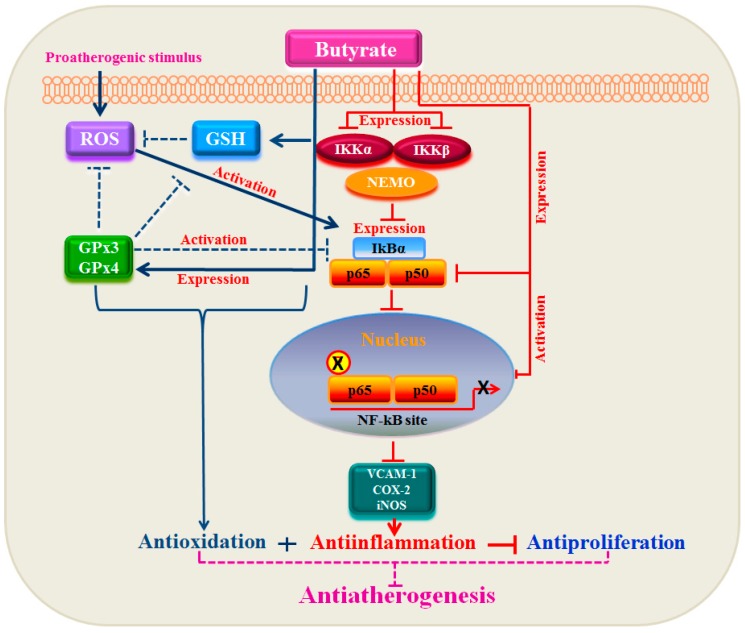
Relationship between GPxs and the NF-κB pathway in the antiproliferation action of butyrate. In response to proatherogenic stimuli, ROS, including hydroperoxides and lipid peroxides, are produced, which activate the redox-sensitive NF-κB signal cascade leading to the expression of target genes. Treatment of VSMC with butyrate causes a strong antioxidant effect by upregulating GPxs along with an increase in the cellular GSH level and upregulation of several isoforms of GSTs (not shown in the scheme), which reduces ROS [12] and blocks the NF-κB cascade early in the pathway. Besides, butyrate appears to inhibit the activation of the NF-κB cascade mainly by inhibiting the synthesis of its core components, which coincides with the inhibition of the activation of the NF-κB cascade. This results in the inhibition of NF-κB target gene expression, causing an anti-inflammatory response. Thus, there is a link between the antioxidant effect and anti-inflammatory response in butyrate-arrested VSMC proliferation, a crucial factor in atherogenesis.

Our present study together with our earlier study [12] collectively demonstrate that butyrate, a natural HDAC inhibitor, exhibits an antioxidant effect by escalating the cellular GSH level, diminishing the ROS level and upregulating several GST isoforms [12], GPx3 and, particularly, GPx4 (Figure 1, Figure 2, Figure 3, Figure 4 and Figure 5), the scavenger of lipid hydroperoxides and other membrane-bound complex hydroperoxides, the mediators of atherogenesis, establishing an association between butyrate-induced antioxidant effect and its antiproliferation action. Furthermore, butyrate-induced antioxidant machinery may dampen the activation of the redox-sensitive NF-κB transcription factor cascade by reducing the ROS level by the upregulation of GPxs (Figure 1, Figure 2, Figure 3, Figure 4, Figure 5 and Figure 6) and GSTs [12]. However, importantly, unlike most inhibitors of the NF-κB pathway, including the majority of the chemopreventive nutraceuticals [54,55,56,57], butyrate inhibits the synthesis of core components of the NF-κB pathway, including IKKα, IKKβ, IkBα and the NF-κBp65 subunit. Accordingly, inhibition of the expression of NF-κB target genes, such as VCAM-1, COX-2 and iNOS, that affect VSMC proliferation through inflammatory mechanism [16,32] concurs with the downregulation of core components of the NF-κB pathway. Thus, by promoting the antioxidant effect and anti-inflammatory response, the cellular activities that have been shown to contribute to the inhibition of VSMC proliferation, butyrate appears to exhibit antiatherogenic potential (Figure 10), which is being explored in an atherogenic animal model.
